# Prevalence of NRT Use and Associated Nicotine Intake in Smokers, Recent Ex-Smokers and Longer-Term Ex-Smokers

**DOI:** 10.1371/journal.pone.0113045

**Published:** 2014-11-18

**Authors:** Lion Shahab, Emma Beard, Jamie Brown, Robert West

**Affiliations:** 1 Department of Epidemiology and Public Health, University College London, London, United Kingdom; 2 Department of Clinical, Educational and Health Psychology, University College London, London, United Kingdom; University of Missouri-Kansas City, United States of America

## Abstract

**Background:**

Nicotine replacement therapy (NRT) is used by smokers wanting to reduce their smoking and to quit. However, there are very little data on nicotine intake associated with NRT use in representative population samples. This study aimed to provide estimates for NRT use and associated nicotine exposure among smokers, recent and longer-term ex-smokers in England, a country with a permissive regulatory regime for nicotine substitution.

**Methods:**

In the Smoking Toolkit Study, a monthly series of representative household surveys of adults aged 16+ in England, current and recent ex-smokers who agreed to be re-contacted were followed up 6 months later and standard socio-demographic and smoking characteristics assessed (N = 5,467, response rate 25.1%). A random sub-sample (N = 1,614; 29.5%) also provided saliva, analysed for cotinine.

**Results:**

The sample followed up was broadly representative of the original sample. At follow-up, 11.8% (95%CI 10.9–12.8, N = 565) of current smokers, 34.8% (95%CI 28.9–41.3, N = 77) of recent (≤3 months) ex-smokers, and 7.8% (95%CI 5.6–10.6, N = 36) of longer-term (>3 months) ex-smokers reported using NRT. Smokers who used NRT had similar saliva cotinine concentrations to smokers who did not use NRT (mean ± sd  = 356.0±198.6 ng/ml vs. 313.1±178.4 ng/ml). Recent ex-smokers who used NRT had levels that were somewhat lower, but not significantly so, than current smokers (216.7±179.3 ng/ml). Longer-term ex-smokers using NRT had still lower levels (157.3±227.1 ng/ml), which differed significantly from smokers using NRT (p = 0.024).

**Conclusions:**

Concurrent use of nicotine replacement therapy while smoking is relatively uncommon and is not associated with higher levels of nicotine intake. Among ex-smokers, NRT use is common in the short but not longer-term and among longer-term users is associated with lower nicotine intake than in smokers.

## Introduction

Harm reduction may be defined as reducing psychological or physiological harm from substance use without complete cessation [1]. In the case of tobacco use, harm reduction can involve the partial substitution of cigarettes with non-combustible forms such as nicotine replacement therapy (NRT) to reduce cigarette consumption or for temporary abstinence. Harm reduction may also constitute the complete and permanent substitution of cigarettes with less harmful products, switching smokers from combustible to non-combustible nicotine delivery devices, including NRT [2].

The rationale for harm reduction with NRT is based on the knowledge that most harm is caused by the burning of tobacco and not nicotine *per se* [3]. There is evidence from both population studies and clinical trials that the use of NRT among current smokers can result in reduced cigarette consumption [4]. Moreover, it is associated with both increased motivation to stop and improved quit rates [1], [5] and does not increase overall nicotine intake [6]. Permanent replacement of cigarettes with NRT among ex-smokers has been shown to result in 40% of baseline levels of nicotine being substituted by nicotine replacement products in clinical trials [7], [8]. Trials have also shown that extended use of NRT by ex-smokers may result in better long-term abstinence rates [9], [10].

For this reason, NRT licensing is being changed to allow its use for harm reduction purposes among current and ex-smokers [11], [12]. Yet, little real world data exist on the impact of NRT use for harm reduction. Most data come from clinical trials which are limited by the fact that trial samples tend to differ from general population samples [13] and that NRT is provided free together with behavioural support which may influence usage patterns. By contrast, most NRT is used without advice and bought over the counter [14] and given the recent proliferation of available products [15], up-to-date information is needed. In the UK, NICE therefore has called for further research in the area of harm reduction [16] as investigating this issue will allow more precise quantification of the likely benefits or harms of substituting cigarettes with NRT among current and ex-smokers.

As a first step in this direction, this report describes the prevalence of NRT use and associated exposure to nicotine in three conditions that it might be used in a general population sample: among current smokers for temporary abstinence or smoking reduction, during a quit attempt by recent ex-smokers or for subsequent maintenance of quitting by longer-term ex-smokers. Although it is unlikely that a substantially increased nicotine intake from NRT would be harmful [17], [18], it clearly is a concern for some people and a potential barrier to effective use of nicotine products [19]. Moreover, the question has been raised whether NRT use perpetuates nicotine dependence [20] and this issue can be addressed by looking at relative exposure to nicotine among ex-smokers using and not using NRT as compared with current smokers. Lastly, given that NRT is mainly used over the counter, focusing on real-life general population data provides the best insight into this topic from a public health perspective.

Specifically, this study aimed to answer the following research questions:

1. What is the prevalence of NRT use among current smokers, recent (≤3 months) and longer-term (>3 months) ex-smokers?
2. What is the nicotine intake associated with NRT use among current smokers, recent (≤3 months) and longer-term (>3 months) ex-smokers?

## Methods

### Study design and sampling

The data come from follow-up waves of the Smoking Toolkit Study (www.smokinginengland.info), which is an ongoing series of cross-sectional household surveys in England designed to provide information about smoking prevalence and behaviour. Each month a new sample of approximately 1,800 adults aged 16 and over completes a face to face computer-assisted survey with a trained interviewer. Current smokers and ex-smokers who have quit within the last year are asked at baseline whether they agree to be followed up and those consenting are re-contacted via a postal questionnaire at 6 months. Half of those who are followed up are randomised to receive also a saliva kit and asked to provide a sample. The survey methodology has been described in detail elsewhere and has been shown to result in a baseline sample that is nationally representative in its socio-demographic composition and proportion of smokers [21]. Participants provided verbal consent. As this was an omnibus household survey conducted every week by the survey company and data were anonymised, written consent was not required. Verbal consent was noted by the interviewer and ethics approval for this study and the consent procedure was granted by the University College London ethics committee.

### Participants

Between November 2006 (the survey start) and July 2011 (when follow-up saliva collection was paused), 21,821 current smokers and recent ex-smokers at baseline agreed to be followed up. Of these, 5,539 responded at 6 months follow-up. Seventy-two participants (1.3%) were excluded due to missing information on NRT use or smoking status which resulted in a response rate of 25.1% and a total analytic sample of N = 5,467, of whom 29.5% (N = 1,614) also provided saliva.

### Measures

At baseline, standard socio-demographic characteristics including age, gender and social-grade (AB  =  higher and intermediate professional/managerial, C1  =  supervisory, clerical, junior managerial/administrative/professional, C2  =  skilled manual workers, D =  semi-skilled and unskilled manual workers, E =  on state benefit, unemployed, lowest grade workers) were assessed. Participants were asked if they (a) smoked cigarettes (including hand-rolled) every day; (b) smoked cigarettes (including hand-rolled) but not every day; (c) did not smoke cigarettes at all but did smoke tobacco of some kind (e.g. pipe or cigar); (d) had stopped smoking completely in the last year; (e) had stopped smoking completely more than a year ago; or (f) had never been a smoker (i.e. smoked for a year or more). Current smokers, classified as answering ‘yes’ to (a) or (b), and recent ex-smokers, classified as answering ‘yes’ to (d), were eligible for follow-up. Those answering ‘yes’ to (c), (e) or (f) were excluded from analysis. Additionally, current smokers were asked questions to determine nicotine dependence (measured by heaviness of smoking index, HSI [22], and strength of urges to smoke, SUTS [23]) as well as motivation to quit (measured by the motivation to stop scale, MTSS [24]).

At 6-months follow-up, all participants were asked whether they smoked cigarettes at all nowadays, including hand-rolled cigarettes (Yes/No). Those who self-classified as smokers were asked whether they were trying to reduce how much they smoked and, if so, whether they used NRT for cutting down and/or temporary abstinence (Yes/No). Those who had stopped smoking were asked how long ago they had stopped smoking, categorised into ex-smokers who had stopped up to three months ago or more than three months ago, whether they had used NRT to help them stop, and if so, whether they still used NRT (Yes/No). We chose this cut-off to distinguish standard from longer-term NRT use because three months is the standard recommendation for treatment length. As postal collection of saliva for cotinine analysis, a reliable marker of nicotine intake, has been shown to be practical and reliable [25], saliva was collected with a postal saliva sample kit at follow-up. The kit contained a salivette cotton roll and instructions on how to collect the sample. Participants then returned the kit by post directly to the laboratory where it was assayed for cotinine using rapid liquid-gas chromatograpy [26].

### Analysis

Data were analysed with IBM SPSS Statistics 20.0.0. Comparisons were made between those who were and were not followed up and among those who were followed-up, between those who did and did not provide a saliva sample. Differences were assessed with χ^2^-tests and independent t-tests for categorical and continuous variables, respectively. Due to the positively skewed distribution of cotinine values, generalised linear models with a gamma distribution and log link were used to determine the impact of NRT use and smoking status on cotinine values. In sensitivity analysis, findings were re-examined with a general linear model using log-transformed cotinine values (all zero values being replaced with 0.001). Given unequal group sizes and non-normality of cotinine values, post-hoc analyses of group differences were assessed with Kruskal-Wallis pairwise comparison. All analyses were unweighted, statistical significance was set at the standard level (p<0.05), and the Bonferroni correction was applied in post-hoc analyses.

## Results

### 1. Prevalence of NRT use among current smokers, recent and longer-term ex-smokers

Participants followed-up at 6 months who constituted the analytic sample were somewhat older and more likely to be female than those lost to follow-up (Table 1). The majority of the analytic sample, 87.5% (95%CI 86.6–88.3, N = 4,783/5,467), were smoking, 4.0% (95%CI 3.6–4.6, N = 221/5,467) had stopped smoking up to three months ago and 8.5% (95%CI 7.8–9.2, N = 463/5,467) more than three months ago. NRT use was most common among recent (≤3 months) ex-smokers, a third of whom (34.8%, 95%CI 28.9–41.3, N = 77/221) were still using NRT. A significantly smaller proportion of current smokers (11.8%; 95%CI 10.9–12.8, N = 565/4,783; χ^2^(1) = 100.2, p<0.001) or longer-term (>3 months) ex-smokers (7.8%; 95%CI 5.6–10.6, N = 36/463; χ^2^(1) = 79.5, p<0.001), were currently using NRT.

**Table 1:** Baseline characteristics by follow-up status and cotinine availability.

	Total sample (N = 21821)	Not followed-up (N = 16354)	Followed-up (N = 5467)	Cotinine analysed	
				Yes (N = 1614)	No (N = 3853)
*Socio-demographic characteristics*					
Mean (SD) Age	41.6 (16.3)	39.8 (16.1)	47.0 (15.6)***	46.8 (15.8)	47.1 (15.5)
% (N) Women	53.1 (11589)	51.9 (8493)	56.6 (3096)***	55.5 (895)	57.1 (2201)
% (N) C2DE^1^	67.8 (14788)	68.1 (11132)	66.9 (3656)	66.7 (1076)	67.0 (2580)
*Smoking characteristics*					
% (N) Current Smokers	93.7 (20445)	94.3 (15426)	91.8 (5019)***	91.5 (1477)	91.9 (3542)
Mean (SD) Heaviness of smoking index∧	2.25 (1.5)	2.22 (1.5)	2.35 (1.5)***	2.41 (1.5)	2.33 (1.5)
Mean (SD) Strength of urges^∧2^	2.29 (0.9)	2.28 (0.9)	2.32 (0.9)**	2.32 (0.9)	2.30 (0.9)
Mean (SD) Motivation to stop^∧3^	3.85 (2.0)	3.92 (2.0)	3.64 (2.0)***	3.67 (2.0)	3.62 (2.0)

1 In socio-economic group C2 (Skilled manual worker), D (Semi-skilled and unskilled manual worker), or E (On state benefit, unemployed, lowest grade workers);

2 From 1 ‘slight’ to 5 ‘extremely strong’;

3 From 1 ‘Don’t want to stop’ to 7 ‘Really want to and intend to stop in next month’,

∧ Only current smokers included;

*p<.05;

**p<.01;

***p<.001.

### 2. Nicotine intake associated with NRT use among current smokers, recent and longer-term ex-smokers

**Figure 1:**
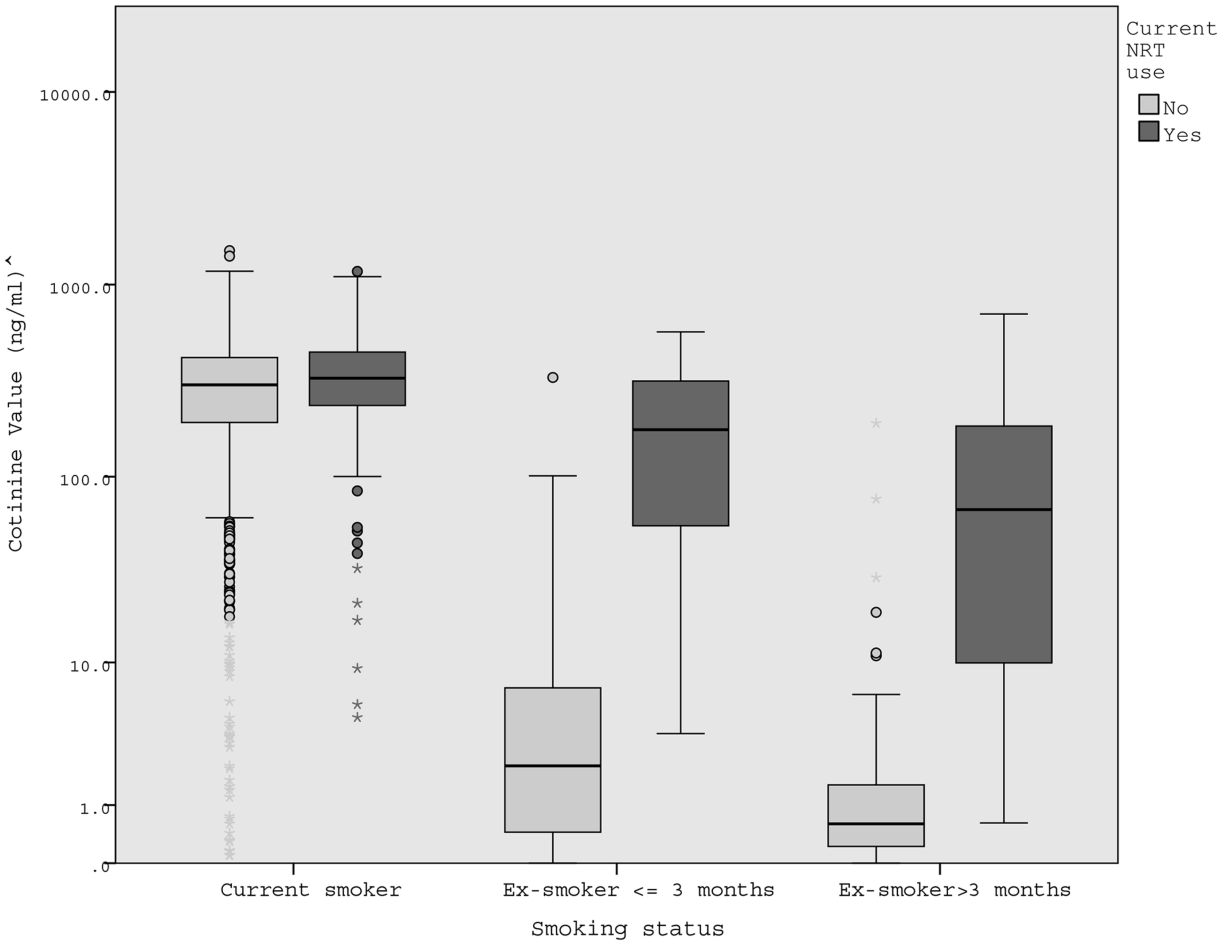
Box-plot of cotinine levels by NRT use and smoking status. Box provides interquartile range and median value is indicated by black line; whiskers represent normal range (up to 1.5 times of interquartile range); circle indicates outliers 1.5–3 times of the interquartile range and asterisks extreme outliers more than 3 times of the interquartile range; ∧Plotted on logarithmic scale.

A subsample of the analytic sample provided a saliva sample, analysed for cotinine to estimate exposure to nicotine. Socio-demographic and smoking characteristics did not differ as a function of whether participants did or did not have cotinine results (all p>0.05, Table 1). In addition, the prevalence of NRT use among either current or ex-smokers did not differ as a function of cotinine availability (all p>0.05). In the presence of a significant interaction between NRT use and smoking status (Wald χ^2^(2) = 55.7, p<0.001), main effects were not considered. As Figure 1 shows, cotinine levels were greatest among current smokers and lowest among longer-term ex-smokers but also differed as a function of NRT use.

Among participants not using NRT, cotinine levels were significantly higher in smokers (arithmetic mean (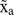) ± sd  = 313.1±178.4 ng/ml, geometric mean (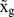) = 226.0 ng/ml, N = 1,263) than ex-smokers. This was the case for both recent ex-smokers (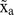 = 16.1±51.1 ng/ml, 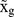 = 1.8 ng/nl, N = 47; Kruskal-Wallis pairwise comparison  = 729.7, p<0.001) and longer-term ex-smokers (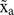 = 3.8±18.8 ng/ml, 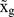 = 0.6 ng/ml, N = 120; Kruskal-Wallis pairwise comparison  = 789.6, p<0.001). Yet, even among ex-smokers there was some variation and some 7.2% (N = 12) had cotinine values above standard cut-off levels for smoking abstinence (≥15 ng/ml), most likely due to misreporting.

Among participants using NRT, cotinine levels of current smokers (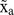 = 356.0±198.6 ng/ml, 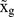 = 283.6 ng/ml; N = 155) were significantly higher only compared with longer-term ex-smokers (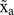 = 157.3±227.1 ng/ml, 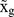 = 34.2 ng/ml, N = 9; Kruskal-Wallis pairwise comparison  = 504.5; p = 0.024). Cotinine levels of recent ex-smokers using NRT (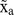 = 216.7±179.3 ng/ml, 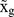 = 113.3 ng/nl, N = 20) did not differ from current smokers using NRT (Kruskal-Wallis pairwise comparison  = 317.0; p = 0.063). Excluding recent ex-smokers who had stopped within the last week (N = 32) did not change results.

Further pairwise comparisons revealed that recent ex-smokers using NRT had significantly higher cotinine levels than recent ex-smokers not using NRT (Kruskal-Wallis pairwise comparison  = 503.0, p<0.001). Longer-term ex-smokers using NRT also appeared to have higher cotinine levels than long-term ex-smokers not using NRT but this difference did not reach significance (Kruskal-Wallis pairwise comparison  = 375.4; p = 0.297). Lastly, smokers with concurrent NRT use had similar cotinine values to those not using NRT (Kruskal-Wallis pairwise comparison  = 90.2; p = 0.344) and this remained the case when controlling for cigarette consumption in sensitivity analysis. Excluding participants who had indicated ever using electronic cigarettes at baseline (N = 9) did not alter results.

## Discussion

Whilst a third of ex-smokers in England use nicotine replacement therapy for smoking cessation in the short-term, its use for harm reduction is relatively uncommon. Only around one in ten smokers uses NRT concurrently and a similar proportion of ex-smokers uses NRT beyond the standard length of three months. Despite recent policy and licensing changes, long-term NRT use does not appear to have increased materially since 2002, when one year usage rates were estimated at around 5% [27]. Similarly, concurrent NRT use among smokers, either for temporary abstinence or cutting down, has remained relatively stable since 2002 [28] and mostly reflects short-term use [29]. These findings are in agreement with a similar lack of change in general NRT usage pattern following an earlier relaxation of NRT licensing in 2005 [30].

Notwithstanding concerns among potential users and stop smoking advisors (e.g. [19]), what little research exists suggests that long-term NRT use is safe and any associated health risks small [17], [31], [32], certainly compared with continued smoking [33]. This study adds further evidence, suggesting that longer-term NRT use is associated with significantly lower exposure to nicotine than among current smokers. By contrast, recent ex-smokers using NRT had concentrations not greatly dissimilar to those of smokers. This finding is consistent with previous clinical studies which show that nicotine substitution from NRT tapers off over time [7]. In line with other work from the Smoking Toolkit Study, NRT use among current smokers was not associated with greater cotinine levels [6], [34], suggesting that smokers are relatively adept at titrating nicotine levels, with some nicotine otherwise obtained from cigarettes being replaced by nicotine from NRT. While this study cannot provide exact estimates of substitution rates as no NRT usage data were available, some substitution is likely given previous findings of smokers maintaining nicotine levels when using acute forms of NRT whilst dramatically reducing cigarette consumption [4]. The fact that longer-term use among ex-smokers was associated with lower cotinine levels suggests that NRT is unlikely to maintain nicotine dependence in the long run. These results should allay the fears of potential NRT users that it will lead to an increase in nicotine exposure.

This study has a number of limitations. Despite an initial large sample size, there were few ex-smokers who used NRT which reduced power to detect differences between groups. In addition, the baseline sample differed somewhat from the sample followed up. However, differences were modest and unlikely to substantially influence the findings. Lastly, due to the cross-sectional design we cannot make causal attributions about the direction of the observed effects. This study's strengths include the use of a general population sample enabling us to look at actual use of NRT and assessment with established, ecologically valid measures. Further research would benefit from measuring a wider array of biomarkers of smoking-related harm such as lung function tests or carcinogen metabolites to complement these results and provide a more complete assessment of the potential harm of long-term NRT use.

In conclusion, use of NRT while smoking is not associated with higher overall nicotine levels; its use for more than 3 months after stopping is uncommon and is associated with significantly lower cotinine levels compared with current smokers.
